# 非手术方法治疗局部侵袭性胸腺肿瘤

**DOI:** 10.3779/j.issn.1009-3419.2016.07.11

**Published:** 2016-07-20

**Authors:** 常禄 王, 兰婷 高, 长兴 吕, 蕾 朱, 文涛 方

**Affiliations:** 1 200030 上海，上海交通大学附属上海胸科医院放疗科 Department of Radiation Oncology, Shanghai Chest Hospital, Shanghai Jiaotong University, Shanghai 200030, China; 2 200030 上海，上海交通大学附属上海胸科医院病理科 Department of Pathology, Shanghai Chest Hospital, Shanghai Jiaotong University, Shanghai 200030, China; 3 200030 上海，上海交通大学附属上海胸科医院胸外科 Deparment of Thoracic Surgery, Shanghai Chest Hospital, Shanghai Jiaotong University, Shanghai 200030, China

**Keywords:** 胸腺肿瘤, 放疗, 化疗, Thymic tumor, Radiotherapy (RT), Chemotherapy

## Abstract

**背景与目的:**

手术切除是早期胸腺肿瘤的主要治疗方法, 而对于Ⅳ期的病变, 化疗则是最常用的方案。对于局部晚期的肿瘤, 尤其是不适合手术的病例, 何种治疗方案效果更优则没有明确的结论。鉴于此, 我们做了这项回顾性的研究, 通过对三种非手术疗法的比较, 希望找到一些线索。

**方法:**

自2000年10月至2010年12月, 共有42例患者接受了三种非手术方案的治疗。这三种模式分别是单独放疗(radiotherapy, RT)、序贯化放疗(sequential chemoradiation, SCRT)以及同步放化疗(concurrent chemoradiation, CCRT)。并对三种方案的缓解率(objective response rate, ORR)、总生存期(overall survival, OS)以及治疗的相关毒副反应进行比较。

**结果:**

全组42例患者中, 总的缓解率为61.9%, 5年生存率为46%。RT组、SCRT组以及CCRT组的缓解率分别是43.8%、50%和87.5%(RT*vs* SCRT, *P*=0.692;RT *vs* CCRT, *P*=0.009;SCRT *vs* CCRT, *P*=0.051)。RT组、SCRT组以及CCRT组的5年生存率分别是30%、50%和61.9%(RT *vs* SCRT, *P*=0.230;RT *vs* CCRT, *P*=0.011;SCRT *vs* CCRT, *P*=0.282)。共有11例患者发生了3度-4度的中性粒细胞减少, 其中7例出现在CCRT组, 另4例出现在SCRT组。有9例患者主诉有3度放射性食道炎, 其中RT组2例, SCRT组3例, CCRT组4例。另外, CCRT组还出现了2例3度的放射性肺炎。未发现致命的5度毒副反应。

**结论:**

在治疗不适合手术的局部晚期胸腺肿瘤上, CCRT显示出了比RT和CCRT更好的局部控制以及长期生存优势, 不过也有增加肺损伤风险的可能。对于局部侵袭性的胸腺肿瘤, CCRT可提供最佳的肿瘤控制效果。

## Abstract (en)

**Background and objective:**

Surgical resection remains the mainstay of treatment for patients with early-staged thymic tumors, while chemotherapy is most commonly used in stage Ⅳ cases.As for locally advanced thymic tumors, especially those unsuitable for surgery, the optimal therapy is still controversial.Thus, we conducted this retrospective study by comparing three nonsurgical treatment modalities to find some clues.

**Methods:**

Three treatment modalities were used in 42 patients from October 2000 to December 2010, including radiotherapy (RT) alone, sequential chemoradiation (SCRT) and concurrent chemoradiation (CCRT).Objective response rate (ORR), overall survival (OS) and toxicity of the three regimens were compared accordingly.

**Results:**

The ORR in all 42 patients was 61.9%, and 5-year OS was 46%.The ORR of RT, SCRT and CCRT were 43.8%, 50% and 87.5%, respectively (RT *vs* SCRT, *P*=0.692;RT *vs* CCRT, *P*=0.009;SCRT *vs* CCRT, *P*=0.051).The 5-year OS of RT, SCRT and CCRT were 30%, 50% and 61.9%, respectively (RT *vs* SCRT, *P*=0.230;RT *vs* CCRT, *P*=0.011;SCRT *vs* CCRT, *P*=0.282).Eleven patients developed neutropenia of grade 3-4, with 7 in CCRT group and 4 in SCRT, respectively.Nine patients experienced esophagitis of grade 3 with 2 in RT, 3 in SCRT and 4 in CCRT.There were also two cases of grade 3 radiation induced pneumonitis in CCRT group.No life-threatening side effects were noted.

**Conclusion:**

When used to treat locally advanced thymic tumors unsuitable for surgery, CCRT performed more favorably than RT alone or SCRT in both tumor response and long time survival, but probably with the increasing risk of pulmonary damage.CCRT may offer the best chance of disease control in the management of locally advanced disease.

胸腺肿瘤是一种起源于胸腺上皮细胞的罕见肿瘤, 其在人群中的发病率约为0.13/100, 000^[1]^。对于肿瘤处于早期的患者, 以手术治疗为主, 其10年生存率约为71%-100%^[2]^。而对于Ⅳ期以上的病变, 则以全身化疗为治疗的基础。对于局部晚期的胸腺肿瘤, 往往先行诱导的化/放疗, 待肿瘤退缩后再行手术切除, 这是因为完整的手术切除已被认为是影响生存的最主要的预后因素^[3]^。然而在实际临床工作中, 总会遇到无法手术切除的情况, 比如肿块广泛侵及临近的重要器官, 或心肺功能较差, 无法耐受手术。对于这类患者, 至今没有一个公认的优化方案。因为该病的低发病率, 也很少有研究去比较不同的非手术治疗方案在这类患者中疗效及安全性的优劣。这里, 我们回顾性分析了42例该类患者, 在10年的时间内, 在单一治疗中心内的治疗经过, 希望对这一类难治性肿瘤在制定优化方案上提供帮助。

## 1 对象和方法

### 1.1 研究对象

自2000年10月至2010年12月61例胸腺肿瘤患者在上海市胸科医院放疗科接受了根治性放疗(radiotherapy, RT)、序贯化放疗(sequential chemoradiation, SCRT)以及同步放化疗(concurrent chemoradiation, CCRT)。其中42例患者被纳入本研究。纳入标准如下:①治疗前经组织学病理证实为胸腺肿瘤; ②根据影像资料判断为侵袭性Ⅲ期病变; ③Ⅳ期病变需满足如下条件:肿瘤旁的胸膜结节转移或淋巴结肿大, 即所有可见病灶均可以包括在一个合理的照射野范围内; ④没有远处器官的转移。所有患者的信息通过病例回顾及电话随访获得。

需要说明的几点是，在2000年到2006年期间，所有的放射治疗均通过三维适形照射来完成，之后逐步被调强放疗的技术所取代。在SCRT治疗中，化疗是作为初始治疗手段，当肿瘤对化疗没有反应或者有缩小且已退缩至稳定的状态，这时放疗参与进来。在CCRT组，放化疗作为初始治疗，在同一天开始。同步治疗完成后是否进行巩固化疗以及化疗的周期数，视患者的具体状况由临床医师决定。根据病例记录，共有16例患者接受单纯放疗，未接受化疗的主要原因是合并了禁忌症（如肾功能不全、高龄、帕金森病等）。

### 1.2 缓解情况及毒副反应的评估

2014年ITMIG (International Thymic Malignancy Interest Group)提出了一套新的针对胸腺肿瘤的实体肿瘤的疗效评价标准(Response Evaluation Criteria in Solid Tumors, RECISTs)^[4]^, 本研究的肿瘤缓解评估据此完成。毒副反应的判断则参照常见治疗不良反应标准(Common Terminology Criteria for Adverse Events, CTCAE 4.0)版本。

### 1.3 统计学方法

采用SPSS 16.0软件进行统计学分析。分类数据的比较使用卡方检验或者*Fisher*精确检验。生存分析采用*Kaplan-Meier*法, 生存率的比较采用*Log-rank*检验。使用*Cox*回归模型进行多因素生存分析, 计算风险比及其95%CI。所有的检验都是双边检验, *P*<0.05为差异有统计学意义。

## 2 结果

共有42例患者被纳入本研究, 患者的详细信息见表 1。其中, 16例患者接受了单独放疗, 10例序贯化放疗, 另有16例接受同步放化疗。中位放疗剂量60 Gy (34 Gy-70 Gy)。在10年的期间内, 化疗的方案有过多种变化, 不过这当中多西他赛联合顺铂的方案(DP)仍是最常用的。具体的化疗方案及应用的次数见表 2。

**1:** 患者的基本信息 Clinical characteristics of patients at baseline

Variables	*n*(%)
Age(yr)	54(17-77)
Gender	
Male	28(66.7)
Female	14(33.3)
Tumor size (cm)	
WHO classification	
B2	4(9.5)
B3	7(16.7)
C	31(73.8)
Masaoka stage	
Ⅲ	35(83.3)
Ⅳa	4(9.5)
Ⅳb	3(7.2)
注：本表得到版权所有者2011-2016 Journal of Thoracic Disease复制许可。	

**2:** 26例接受化疗患者使用过的方案 Chemotherapy regimen and cycles used in 26 patients

Treatment	Regimen	Cycles(*n*)
Concurrent	DP	20
	CAP	2
Sequential	DP	19
	IVP	11
	CAP	6
	MVP	3
	NP	1
DP:docetaxel+cisplatin; CAP:cyclophosphamide+doxorubicin+cisplatin; IVP:ifosfamide+etopiside+cisplatin; MVP:mitomycin+vindesine+cisplatin; NP:vinorelbine+cisplatine. 注:本表得到版权所有者©2011-2016 Journal of Thoracic Disease复制许可。		

### 2.1 缓解的情况及生存分析

42例患者中总的ORR为61.9%(26/42)。不同治疗方案的ORR情况见表 3, CCRT组的ORR高于RT组(87.5%*vs*43.8%, *P*=0.009)和SCRT组(50%, *P*=0.051)。在SCRT组中, 亚组分析比较了DP方案和非DP方案缓解率的差异, 结果显示无明显差异(75%*vs*50%, *P*=0.571)。

**3:** 不同亚组中的缓解率情况 The overall response rate in different subgroups

Subgroup	*n*	ORR(%)	*P* value
Treatment modality				
RT^1^	16	43.8	1 *vs* 2=0.692
SCRT^2^	10	50	2 *vs* 3=0.009
CCRT^3^	16	87.5	1 *vs* 3=0.009
Histology type			0.10
Thymoma	11	81.8	
Thymic carcinoma	31	54.8	
Masaoka stage			0.05
Stage Ⅲ	35	68.6	
Stage Ⅳ	7	28.6	
RT:radiotherapy; SCRT:sequential chemoradiation; CCRT:concurrent chemoradiation; ORR:overall response rate. 注:本表得到版权所有者©2011-2016 Journal of Thoracic Disease复制许可。			

全部患者的中位生存期为41个月(40.5-64.5), 5年生存率为46%(图 1)。不同亚组的生存曲线参见图 2-图 4。

**1:**
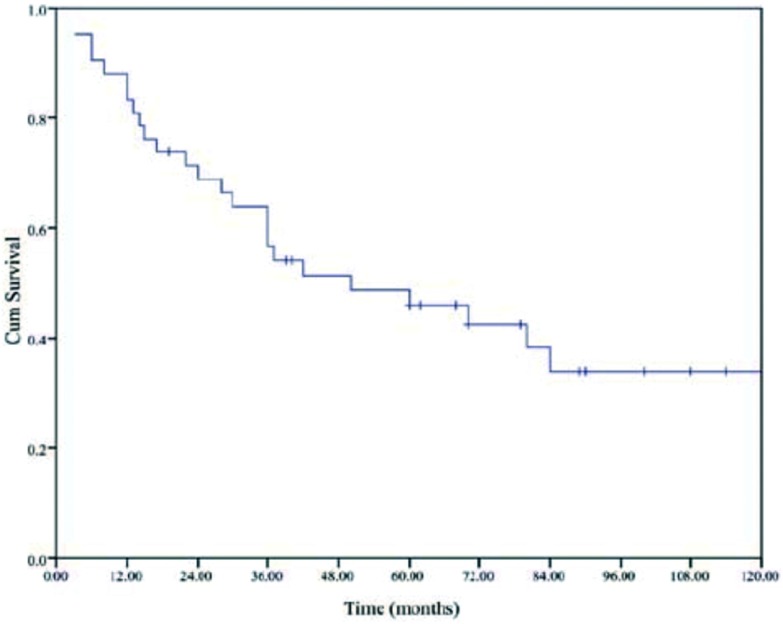
全组42例患者的生存曲线 Overall survival of all 42 patients

**2:**
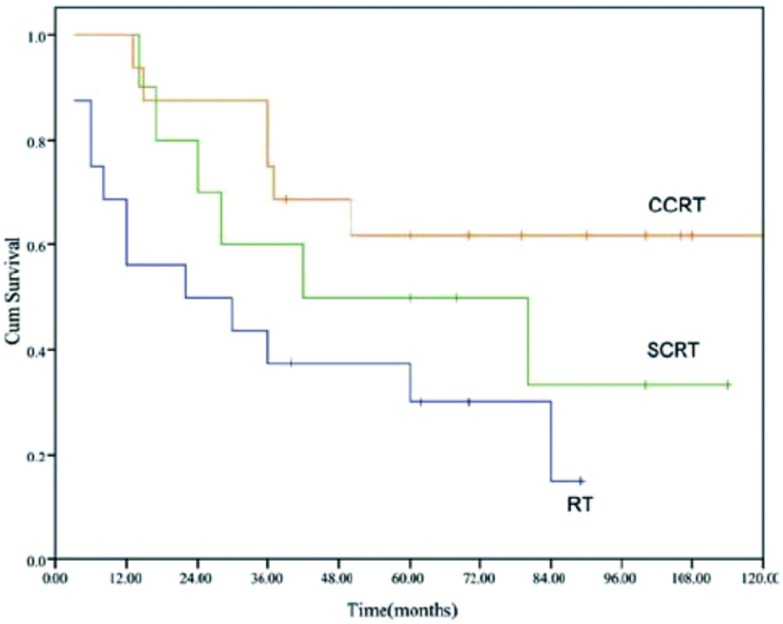
三种不同治疗方法的生存曲线(CCRT *vs* SCRT, *P*=0.282;CCRT *vs* RT, *P*=0.011;SCRT *vs* RT, *P*=0.230) Survival curves of three treatment regimens (CCRT *vs* SCRT, *P*=0.282;CCRT *vs* RT, *P*=0.011;SCRT *vs* RT, *P*=0.230)

**3:**
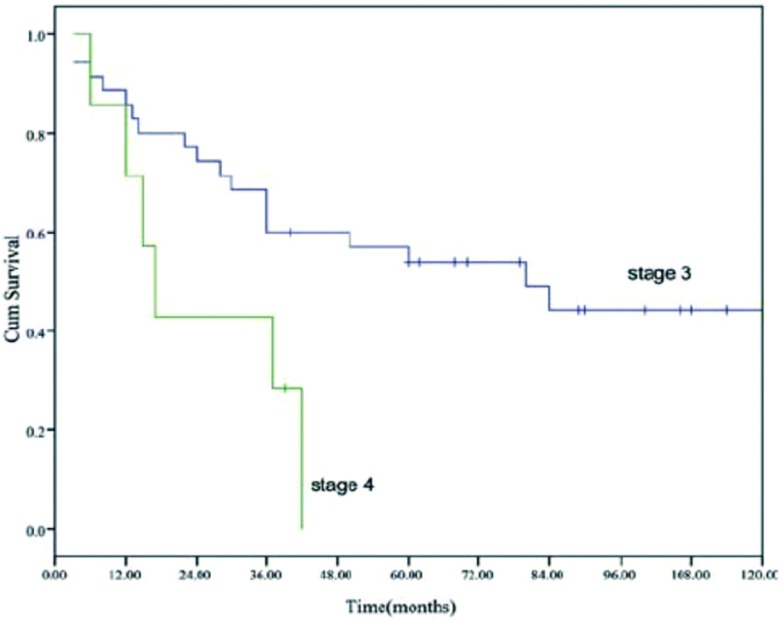
Masaoka Ⅲ期和Ⅳ期的生存曲线(*P*=0.009) Overall survival of patients with Masaoka Ⅲ and Ⅳ tumors (*P*=0.009)

**4:**
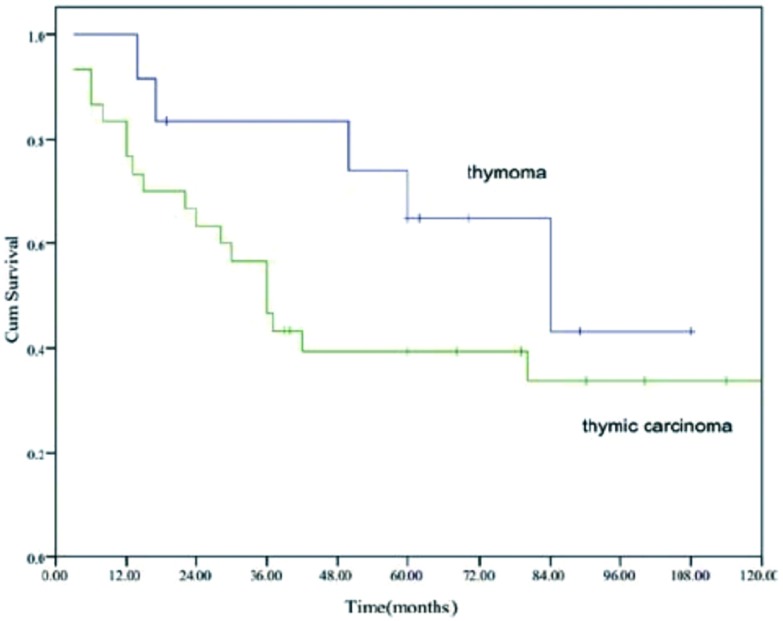
胸腺瘤和胸腺癌的生存曲线(*P*=0.163) Overall survival of patients with thymoma and thymic carcinoma (*P*=0.163)

单因素分析中(表 4), 年龄(*P*=0.031)、Masaoka分期(*P*=0.009)和不同的治疗方案(*P*=0.031)是影响生存期的显著因素, 其中CCRT组的生存期最长。多因素分析则提示Masaoka分期、治疗方案和病理分型(瘤*vs*癌)是独立的预后因子(表 5)。

**4:** 影响生存的单因素分析 Univariate analysis of factors influencing survival

Variables	*P* value
Gender	0.673
Age (<60 yr *vs* ≥60 yr)	0.031
Tumor diameter(<6 cm *vs* ≥6 cm)	0.243
Histology (Thymoma *vs* Carcinoma)	0.163
Masaoka stage (Ⅲ *vs* Ⅳ)	0.009
Treatment (CCRT *vs* Others)	0.031
Radiation dose (<60 Gy *vs* ≥60 Gy)	0.125
注：本表得到版权所有者©2011-2016 Journal of Thoracic Disease复制许可。	

**5:** 影响生存的多因素分析 Multivariate analysis for factors predicting survival

Variables	Hazard ratio (CI)	*P* value
Age(<60 yr *vs* ≥60 yr)	0.818(0.333-2.012)	0.662
Histology	3.465(1.042-11.526)	0.043
Masaoka stage(Ⅲ *vs* Ⅳ)	3.772(1.277-11.139)	0.016
Treatment(CCRT *vs* Others)	0.185(0.054-0.643)	0.008
注：本表得到版权所有者©2011-2016 Journal of Thoracic Disease复制许可。		

### 2.2 毒副反应

全组病例中未出现治疗相关的死亡事件。最主要的毒副反应为3度-4度的中性粒细胞减少, 共出现于11例患者中。其他毒副反应的发生情况见表 6。SCRT组与CCRT组中副反应的发生概率相似(70.0%*vs* 80.3%), 均高于RT组的发生率(12.5%)。

**6:** 不同治疗方式下出现3度-4度副反应的情况 Toxicities of grade 3-4 in different groups

Toxicity	RT(%)	SCRT(%)	CCRT(%)	*P* value(SCRT *vs* CCRT)
Neutropenia	0	4(40)	7(43.8)	0.847
Esophagitis	2(12.5)	3(30)	4(25)	0.783
Pneumonitis	0	0	2(12.5)	0.249
注：本表得到版权所有者©2011-2016 Journal of Thoracic Disease复制许可。				

## 3 讨论

对于包膜完整的、非侵袭性的胸腺肿瘤, 完整切除通常能达到根治效果, 局部复发率小于2%^[2]^。然而对于进展期的胸腺肿瘤, 除手术之外, 尚无标准的治疗规范。在临床工作中, 究竟采用放疗、化疗抑或放化疗联合的手段, 往往取决于肿瘤科医生的临床经验或个人偏好。据我们所知, 该研究是第一次在无法手术的胸腺肿瘤患者中, 比较了3种不同的非手术方案的优劣。我们的结果提示了, CCRT方案无论在近期的肿瘤缓解率以及长期生存率上均取得了优于SCRT和RT的结果。

对于胸腺肿瘤来说, 除了彻底手术切除这一因素, WHO分型和Masaoka分期也被证明是重要的预后影响因子^[2, 5]^。在本研究中, 通过多因素分析, 我们发现除了上述这些因素, 治疗方法也对预后有重要影响(表 5)。与其他2种方法相比, CCRT在ORR上表现出了明显的优势(CCRT *vs* SCRT:87.5%*vs* 50%, *P*=0.051)。当放疗和化疗同时应用时, 两种方法往往表现出协同作用, 能最大程度的杀灭肿瘤细胞。同步放化疗的优越性主要基于如下的机制:①肿瘤组织具有异质性, 细胞成分多样, 化疗药物和放疗结合可以涵盖更多的肿瘤成分; ②局部疗法结合全身疗法, 具有空间协同作用; ③处于不同细胞周期的肿瘤细胞, 对治疗的敏感性不同, 例如S期的细胞对放疗抗拒, 但对化疗药物敏感, 两者同时合用, 大大增强抗瘤效果; ④某些化疗药物, 例如顺铂、紫杉类药物等, 具有放疗增敏作用^[6-9]^。到目前为止, 还没有关于CCRT治疗局部晚期胸腺肿瘤的大规模报道。Chen等^[10]^在16例不能手术的胸腺癌患者中进行了CCRT治疗, 其5年生存率为67.7%, 与我们的研究结果相似(61.9%)。即使与某些以手术为主的治疗^[11-13]^相比(5年生存率约35%), 这些结果也是相当不错的。Wright^[14]^和Korst^[15]^都尝试用CCRT作为诱导手段再辅以手术来治疗局部侵袭性的胸腺肿瘤, 根据术后的病理检验, R0切除率达到了80%, 标本中病理完全缓解率也达到了20%, 这些结果均优于其他的一些术前诱导方案^[16-18]^。因此, 可以开展更大规模的研究来验证CCRT作为诱导治疗在潜在性无法切除的胸腺肿瘤上的作用。

由于是回顾性的研究, 化疗方案之间存在较大差异。但需要说明的一点是, 在CCRT组中, 使用最多的是多西他赛联合顺铂(DP)的方案(91%)。在Chen的研究中^[10]^, ORR率只有50%, 远低于本研究中87.5%的ORR率。进一步分析其中的细节可以发现, 在两个研究中, 中位放疗剂量几乎相同(60 Gy), 但Chen的研究中使用的化疗方案为5-FU联合顺铂, 与我们的DP方案不同。在Wright^[14]^和Korst^[15]^的研究中, ORR率都在45%左右, 也低于我们研究中的缓解率。诚然上述2项研究中给予的放疗剂量为诱导剂量(45 Gy), 低于我们的根治性剂量, 但也应该注意到其中化疗方案的差别(EP *vs* DP)。Watanabe等^[19]^报道了多西他赛单药用来治疗胸腺癌, 也能取得31%的ORR率。在我们的SCRT治疗组中, 我们进行了一个亚组比较, 发现DP方案与其他非DP方案相比, 在肿瘤的缓解率上无明显差异(75%*vs* 50%, *P*=0.571)。即使这样, 在CCRT治疗模式中, DP方案所起的作用也可能有区别, 因为紫杉类药物跟顺铂都具有放疗增敏作用。至少从我们的研究中可以观察到, 同步使用DP化疗与放疗, 可以最大程度的缩小肿瘤体积。鉴于此, 我们需要设计前瞻性的试验来进一步验证DP方案在同步放化疗中的作用。

在毒副反应方面, 未出现致死性的事件。中性粒细胞减少以及放射性食道炎是最常见的两类反应, 但多数症状轻微且易控制。需要说明的一点是, 几乎所有的食管炎均出现在2006年之前, 那时的放射技术主要是三维适形, 而且经常用到前后对穿野。随着照射技术的改进, 调强放疗被越来越多地使用, 就再未出现过严重的食管反应。在CCRT组和SCRT组中, 总体副反应的发生率大致相仿, 然而有2例3度的放射性肺炎均出现在CCRT组中, 提示在进行根治性的CCRT治疗时, 有潜在的肺损伤风险。

当然, 该研究也存在一些不足, 例如病例数少, 回顾性而非前瞻性, 治疗方案不统一, 化疗方案不一致等等。尽管如此, 我们还是发现, 联合DP化疗的CCRT方案, 在肿瘤的缓解率和患者的生存期上均取得了很大的改善。我们认为需要进一步的前瞻性试验来检验这一治疗模式。

总的来说, 在治疗不适合手术的局部晚期胸腺肿瘤上, CCRT与RT或SCRT相比无论在肿瘤缓解率抑或远期生存率上均显示出优势, 但也有可能增加肺损伤的风险。基于以上结果, CCRT可以为此类患者提供最佳的疾病控制机会。另外, 也应该尝试CCRT作为诱导治疗的模式, 通过提高完整切除的几率, 进而改善患者的长期生存。
